# Host trait combinations drive abundance and canopy distribution of atmospheric bromeliad assemblages

**DOI:** 10.1093/aobpla/plw010

**Published:** 2016-02-17

**Authors:** Cleber Juliano Neves Chaves, Júlio César Dyonisio, Davi Rodrigo Rossatto

**Affiliations:** 1 Programa de Pós-graduação em Ecologia e Biodiversidade, Departamento de Ecologia, Instituto de Biociências, Univ. Estadual Paulista, Campus de Rio Claro, 13506-900 Rio Claro, São Paulo, Brazil; 2 Departamento de Biologia, Faculdade de Ciências Agrárias e Veterinárias, Univ. Estadual Paulista, Campus de Jaboticabal, 14884-900 Jaboticabal, São Paulo, Brazil

**Keywords:** Atmospheric bromeliads, canopy ecology, epiphyte assemblage, functional ecology, host preference, phorophyte, *Tillandsia*

## Abstract

Epiphytes are strongly dependent on the conditions created by their host's traits and a certain degree of specificity is expected between them, even if these species are largely abundant in a series of tree hosts of a given environment, as in the case of atmospheric bromeliads. Despite their considerable abundance in these environments, we hypothesize that stochasticity alone cannot explain the presence and abundance of atmospheric bromeliads on host trees, since host traits could have a greater influence on the establishment of these bromeliads. We used secondary and reforested seasonal forests and three distinct silvicultures to test whether species richness, phylogenetic diversity and functional diversity of trees can predict the differential presence, abundance and distribution of atmospheric bromeliads on hosts. We compared the observed parameters of their assemblage with null models and performed successive variance hierarchic partitions of abundance and distribution of the assemblage to detect the influence of multiple traits of the tree hosts. Our results do not indicate direct relationships between the abundance of atmospheric bromeliads and phylogenetic or functional diversity of trees, but instead indicate that bromeliads occurred on fewer tree species than expected by chance. We distinguished functional tree patterns that can improve or reduce the abundance of atmospheric bromeliads, and change their distribution on branches and trunk. While individual tree traits are related to increased abundance, species traits are related to the canopy distribution of atmospheric bromeliad assemblages. A balance among these tree functional patterns drives the atmospheric bromeliad assemblage of the forest patches.

## Abstract (precis)

Anyone interested in plants may already have asked themselves why some trees host a wide diversity and large numbers of epiphytes while others have none. This may occur by chance or could be derived from specific patterns driven by tree traits. Here we evaluated how individual and species characteristics affect the number and location of atmospheric bromeliads. We found that the presence of bromeliads is not stochastic, and that tree traits have a great influence on the presence and abundance of bromeliad species. Additionally, trees can be categorized as worse or better hosts to shelter these plants based on their canopy and trunk characteristics.

## Introduction

About 9 % of vascular plants (nearly 30 000 species) are mechanically dependent on other plants (Benzing 1990; Zotz 2013), and for this reason, they are important elements of many ecosystems, particularly in the neotropics. The epiphytic assemblages provide a remarkable system to evaluate species-specific interactions in plants, since their hosts are capable of facilitating or limiting their fixation and reproduction (Callaway *et al*. 2002; Steel and Wilson 2003; Wagner *et al*. 2015). Given the particularity of each host tree and the niche differences of epiphytic species, a certain degree of specificity is expected between them; i.e. the exclusive occurrence of an epiphyte species on a single host species (ter Steege and Cornelissen 1989; Tremblay *et al*. 1998). Although a strict host tree specificity or a completely random host tree selection is rare, many works report the existence of host preference (i.e. a greater abundance of a epiphyte species on a few host species; e.g. ter Steege and Cornelissen 1989) and host limitation (i.e. a concentration of individuals of a epiphyte species on a few host species as a result of limiting factors of the other tree species; e.g. Vergara-Torres *et al*. 2010) in epiphyte communities.

In both host preference or host limitation scenarios, the host traits play a crucial role on epiphyte assemblage and distribution, since each host tree species offers a specific combination of phenological patterns, architectural traits and bark characteristics that provide a wide spectrum of epiphyte habitats (Zotz and Schultz 2008; Benavides *et al*. 2011). Traits of hosts can propitiate or limit the presence of epiphytes: for example, larger trees have greater available surface and more complex structures to receive epiphyte seeds (e.g. Benzing 1990; Bernal *et al*. 2005; Flores-Palacios and Garcia-Franco 2006; Laube and Zotz 2006*a*, *b*; Benavides *et al*. 2011), trees with rough and furrowed barks can increase seed establishment and improve water absorption (e.g. Callaway *et al*. 2002; Cascante-Marín *et al*. 2009; Wagner *et al*. 2015) and deciduous canopies can facilitate the establishment of more drought-adapted species and limit the success of shade-adapted species (e.g. Cardelus 2007; Einzmann *et al*. 2015). Thus, tree species that are functionally similar and/or phylogenetically related could host similar epiphyte species.

Highly drought-specialist epiphytes are the atmospheric bromeliads, a group composed mainly by species of the *Tillandsia* genera (Bromeliaceae) (Benzing 2012). These plants are often small, absorb water and nutrients almost entirely from aerosols through their squamous leaves (Martin and Schmitt 1989), require high light irradiances and have a crassulacean acid metabolism (Benzing 1981, 1990). Thus, they are found profusely on drier habitats such as dry forests (e.g. Benzing 2000; Reyes-García *et al*. 2008; Flores-Palacios *et al*. 2015), appearing even in outer canopies of wet forests (e.g. Pittendrigh 1948; Johansson 1974; Griffiths and Smith 1983; Einzmann *et al*. 2015). Atmospheric bromeliads can also be largely abundant on trees of anthropogenic transformed habitats, such as silviculture forests, and even on abiotic substrates, such as telephone wire cables and power lines (e.g. Martin *et al*. 1986; Wester and Zotz 2010; Benzing 2012). This creates an assumption that these plants should be able to colonize any substrate (Callaway *et al*. 2002; Wester and Zotz 2010), but their abundances will probably be fostered by substrates that optimize or limit their growth and reproduction (Bernal *et al*. 2005; Valencia-Díaz *et al*. 2010; Vergara-Torres *et al*. 2010). Furthermore, the atmospheric bromeliad preferences could be hampered in an environment with many tree species having distinct facilitator and limiter characters, since it is probable that some traits of a tree, such as crown shape or leaf area, can affect the hosting ability of its neighbour trees.

Here, we used patches of secondary and reforested tropical forests to test whether species richness, phylogenetic relatedness and functional diversity of trees (not only the hosts) can predict the differential presence and abundance of atmospheric bromeliad assemblages on trees. Additionally, we tested which combination of functional traits of the trees of those patches, adding up the trees from three distinct silvicultures (*Pinus elliottii*, *Eucalyptus* spp. and *Tabebuia* spp.), could: (i) facilitate or limit the establishment of atmospheric bromeliads and (ii) predict their distribution on tree hosts (i.e. if individuals attach more to the branches or to the trunk of the trees). Finally, we assessed how the presence or absence of these trait combinations in all studied vegetation patches will affect the whole atmospheric bromeliad assembly. We hypothesized that stochasticity alone cannot explain the presence and abundance of atmospheric bromeliads on host trees, since the assumed microclimatic conditions influenced by host traits may have a greater influence on the establishment and growth of atmospheric bromeliads.

## Methods

### Study site

We performed this study during November 2013 in five vegetation patches differing in species composition at Faculdade de Ciências Agrárias e Veterinárias (FCAV), UNESP, Jaboticabal – SP, Brazil (48°17′S, 21°14′W) **[see Supporting Information—Fig. S1]**. This region has a smooth and wavy relief, ∼600 m above sea level and a typical tropical climate (Souza *et al*. 2003), with an annual rainfall ∼1420 mm (historic average between 1971 and 2000). Our chosen vegetation patches were two diverse forests (mixed-species): a semi-deciduous secondary forest (SF) and a semi-deciduous forest reforestation patch (RP), and three different silviculture forests: a *Eucalyptus* sp. patch (EP), a *P. elliottii* patch (PP) and a *Tabebuia* sp. (Bignoniaceae) grove (TP). The atmospheric bromeliad composition measured on silviculture patches can be attributed to a single functional tree pattern, once, unlike the mixed-species patches (secondary and reforested forest patches), each of them represents an isolated functional group of trees. According to FCAV historical records, these patches were planted around 1979–80, with no management thereafter. In each of these vegetation patches, we randomly assembled five 10m × 10m plots to describe vegetation composition and distribution on canopy **[see Supporting Information—Fig. S2]**. All vegetation patches are spaced <1 km apart; thus, in our study area, the supply and interchange of propagules among vegetation patches can be very high, which makes the disjoint distribution and abundance patterns of atmospheric bromeliads among trees probably caused by their host preferences.

### Data collection

We identified the species and measured trunk diameter at breast height (DBH) and height of each studied tree individual found in each plot. Additionally, we measured the leaf area index (LAI) of each studied plot taking hemispheric photographs with a CI-110-24P-ID (CID Bioscience Inc., Camas, WA, USA). Functional traits related to the capability to host epiphytes (i.e. deciduousness, bark type, peeling capability, heliophyte or esciophyte and presence of thorns and needles; classification based on Lorenzi and Gonçalves 2011) were assessed for each tree species, consulting the literature (Lorenzi et al. 2009; [Bibr PLW010C41][Bibr PLW010C42], *b*). On each tree, of each plot, we counted and identified all atmospheric bromeliads, identifying where they were located (i.e. trunk or branches) **[see Supporting Information—Fig. S2]**.

### The distribution of atmospheric bromeliad assemblage on tree canopies

To analyse the distribution of atmospheric bromeliad assemblages on tree canopies (AB_dst_), we employed the followed metric: AB_dst_ = *A*_trunk_/*A*_total_, where *A*_trunk_ represents the atmospheric bromeliad abundance (AB_abund_) at the trunk and *A*_total_ the total abundance in each host tree. Thus, the closer AB_dst_ is to 1, the more relative abundance of atmospheric bromeliads a tree has on its trunk, and the closer AB_dst_ is to 0, the more relative abundance of atmospheric bromeliads a tree has on its branches. An AB_dst_ closer to 0.5 implies a relative balance between the AB_abund_ on trunk and branches.

### Statistical analysis

To test the differences between AB_abund_ and distribution on tree canopies (AB_dst_) among vegetation patches (i.e. EP, PP, TP, RP and SF; see ‘Study site’ for abbreviations), we used general linear models (GLMs) and analysis of variance (ANOVA), followed by pairwise means comparisons, using *χ*^2^ values for Poisson (count data) and Binomial (proportion data) types of distribution (Crawley 2002). To test the relationship of AB_abund_ and AB_dst_ with tree richness, tree functional diversity (FDi; Petchey and Gaston 2006; Cianciaruso *et al*. 2009) and tree phylogenetic diversity (PD; Faith 1992), we fit simple linear models and tested them also with ANOVA.

To test whether host tree species richness, PD (Faith 1992) and functional diversity (FDi; Cianciaruso *et al*. 2009) of the vegetation patches with diverse tree species (RP and SF) are less, greater or equal to the expected by chance (see Laube and Zotz 2006*b*), we randomized all atmospheric bromeliad individuals raised in each of these vegetation patches on all tree hosts of each environment. Then we calculated the species richness, PD and FDi of host trees on the random draw. We repeated this process 10 000 times, on a specific R language script **[see Supporting Information—File S1]**, generating a null model with a 95 % confidence interval for each of the parameters that were used to compare with the observed values.

To test whether the pool of host tree species raised on both RP and SF have a random phylogenetic structure, we compared the observed phylogenetic structure (correlation between co-occurrence and phylogenetic distance) with patterns expected under a null model (1000 runs, shuffling phylogeny tip labels), based on Schoener's index of co-occurrence (Cavender-Bares *et al*. 2006). In addition, to test whether the pool of host tree species of RP and SF are phylogenetically underdispersed (i.e. if atmospheric bromeliads attach to trees of more restricted phylogenetic groups), we randomized 1000 times their data matrix abundances (maintaining the tree species occurrence frequencies) and compared the observed PD (i.e. phylogenetic species variability, richness, evenness and clustering; Helmus *et al*. 2007) with the null model obtained. To calculate the phylogenetic parameters, we constructed a phylogeny **[see Supporting Information—Fig. S3]** with all tree species through the mega phylogenetic tree of phylomatic software (R20120829; phylodiversity.net/phylomatic).

In order to detect which of the functional traits of trees were more important and how they interact to explain the observed variation in AB_abund_ and AB_dst_, we constructed data trees through successive hierarchic partitions and 1000 randomizations (to test the significance) of all data using: (i) non-host and host trees for AB_abund_ and (ii) only the host trees for AB_dst_. We did so once this second analysis used the percentage of atmospheric bromeliad distribution on each tree canopy and, thus, required that they hosted at least one of those epiphytes. The same procedure was repeated with the subsequent groups generated by the most significant variable until no significant partition could be observed, pulling out all variables that already had explained the variance of the previous partitions. To split groups when the most explanatory variable was quantitative, we divided the data into two groups by their median. With the resulting data tree, we traced how the traits of trees interact to increase or decrease the abundance and structuring of atmospheric bromeliad assemblage. Additionally, we determined which patterns of interactions among functional traits of trees (host patterns) are more prone to (i) host larger numbers (higher than 400) of atmospheric bromeliads (named here as ‘best hosts’), (ii) host fewer numbers (<10) of atmospheric bromeliads (named as ‘worst hosts’), (iii) host >90 % of atmospheric bromeliads on the trunk (named as ‘trunk hosts’) and (iv) host >90 % of atmospheric bromeliads on the branches (named as ‘branches hosts’).

The relationships among the assignment of each tree to each Host pattern were tested through GLMs. The correlations among these assignments were grouped in only one variable, through a principal component analysis (Hill and Smith 1976) of (i) all trees and (ii) only the host trees, to test relationships of the Host patterns with, respectively, (a) AB_abund_ and (b) AB_dst_, through GLMs. Finally, we performed an Euler diagram to observe whether some species have tree individuals assigned to different Host patterns and whether some species have tree individuals assigned exclusively to a single Host pattern. All the analyses were performed on R software 3.1.2 (R Development Core Team 2014), through ‘vegan’, ‘picante’, ‘rich’, ‘FD’, ‘ade4’, ‘phytools’, ‘hier.part’ and ‘venneuler’ packages (Dray and Dufour 2007; Kembel *et al*. 2010; Wilkinson 2011; Revell 2012; Walsh and Mac Nally 2013; Laliberté *et al*. 2014; Oksanen *et al*. 2015).

## Results

### Atmospheric bromeliad species and differences among vegetation patches

We found four species of atmospheric bromeliads at our patches of sampled vegetation: *Tillandsia recurvata*, *T. pohliana*, *T. tricholepsis* and *T. loliacea* **[see Supporting Information—Fig. S4]**. *Tillandsia recurvata* was the most abundant species (Table 1), representing >80 % of the atmospheric bromeliads found in our study area (57.6 % at EP, 82.1 % at TP, 69.7 % at PP, 81.5 % at RP and 52.5 % at SF).

**Table 1.** Abundances of atmospheric bromeliad species found on each vegetation patch.

Vegetation patch	*Tillandsia recurvata*	*Tillandsia pohliana*	*Tillandsia tricholepsis*	*Tillandsia loliacea*
*Eucalyptus* sp. (EP)	76	40	7	9
*Tabebuia* spp. (TP)	15 927	207	336	207
*Pinus elliottii* (PP)	570	159	17	72
Reforestation (RP)	44	5	0	5
Secondary forest (SF)	52	40	4	3
Total	16 669	451	364	296

**Figure 1.**
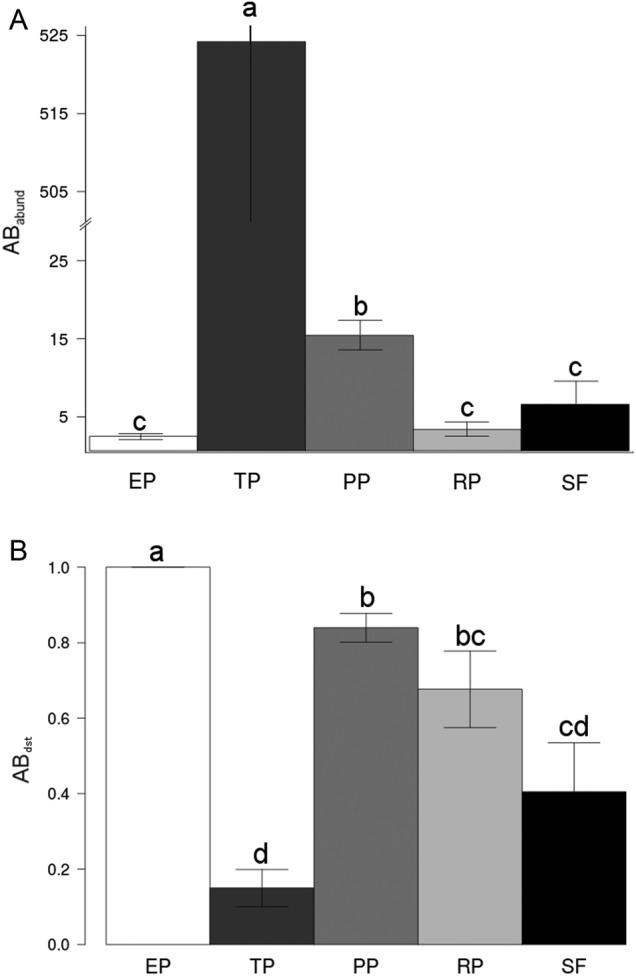
(A) Abundance and (B) distribution on tree canopies of atmospheric bromeliad assemblages along the tree hosts of EP, TP, PP, RP and SF. The closer AB_dst_ is to 1, the greater the abundance of atmospheric bromeliads on the trunk, and the closer AB_dst_ is to 0, the greater the abundance on branches. Vertical bars indicate standard error of means. The same letters indicate statistical similarity between vegetation patches according to Tukey's test (*P* = 0.05).

The *Tabebuia* spp. patch had by far the greatest AB_abund_ by host [*χ*^2^ = 52 609, df = 4, *P* < 0.001; see Fig. 1A and also **Supporting Information—Fig. S5**], which was distributed primarily on branches (*χ*^2^ = 97.14, df = 4, *P* < 0.05; Fig. 1B). On the other hand, the trees of EP presented the lowest average number of atmospheric bromeliads by host (*P* < 0.05; Fig. 1A) with all of them attached to the trunk (Fig. 1B). We found no statistically significant relationships of AB_abund_ and AB_dst_ with species richness, functional diversity or PD of the trees (*P* > 0.05). While almost all trees of TP and PP served as host for atmospheric bromeliads, >60 % of trees in the RP and SF showed no epiphytes **[see Supporting Information—Fig. S6]**.

### Tree species richness, phylogenetic and functional diversity on atmospheric bromeliad assembly

**Figure 2.**
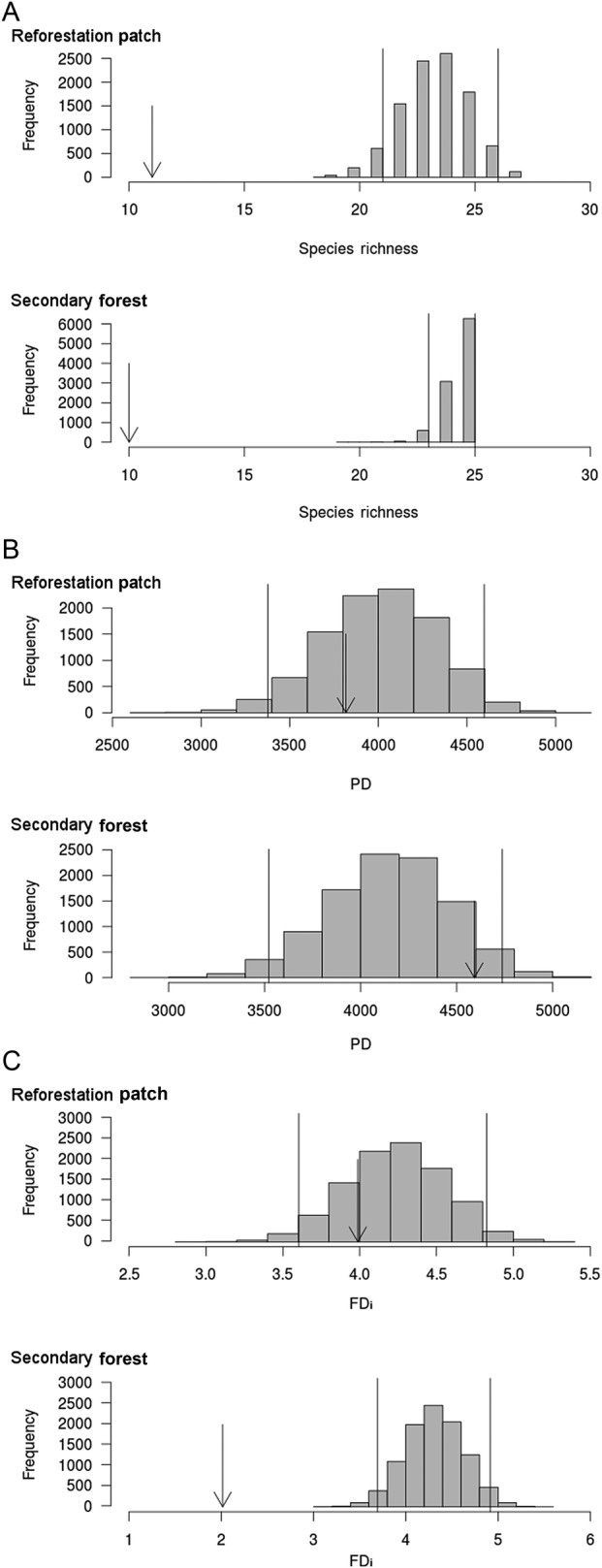
Histograms of (A) tree species richness, (B) PD and (C) functional diversity (FDi) of atmospheric bromeliads on each sampled tree on reforestation and SF patches. The vertical lines delimit the 95 % confidence intervals of the obtained null models. The vertical arrows point to the observed values of each index.

Based on 95 % confidence intervals, the host species richness in the RP and SF, as well the functional diversity (FDi) of SF, were lower than expected by chance (Fig. 2). However, the PD of both vegetation patches and functional diversity (FDi) of RP was similar to null models (Fig. 2). The host tree species raised on RP and SF have a random phylogenetic structure, since we observed no difference between the observed phylogenetic structure and the null model. Furthermore, the phylogenetic species diversity (i.e. phylogenetic species variability, richness, evenness and clustering) of RP and SF also showed a random or even an overdispersed phylogenetic structure (based on 95 % confidence intervals; Table 2).

**Table 2.** Comparison between the observed and the randomized (95 % confidence interval) phylogenetic structure of the host trees of RP and SF with its conclusive inference on distinct PD metrics. PSV, phylogenetic species variability; PSR, phylogenetic species richness; PSE, phylogenetic species evenness; PSC, phylogenetic species clustering.

Metric	Vegetation patch	Null model quantiles		Observed	Phylogenetic structure
		2.5 %	97.5 %		
PSV	RP	0.741	0.914	0.831	Random
	SF	0.702	0.765	0.717	Random
PSR	RP	2.585	3.777	3.754	Random
	SF	2.134	2.962	3.585	Overdispersed
PSE	RP	0.651	0.869	0.757	Random
	SF	0.508	0.718	0.635	Random
PSC	RP	0.151	0.372	0.284	Random
	SF	0.267	0.364	0.386	Overdispersed

### Functional patterns of the host trees affecting bromeliad assembly

**Figure 3.**
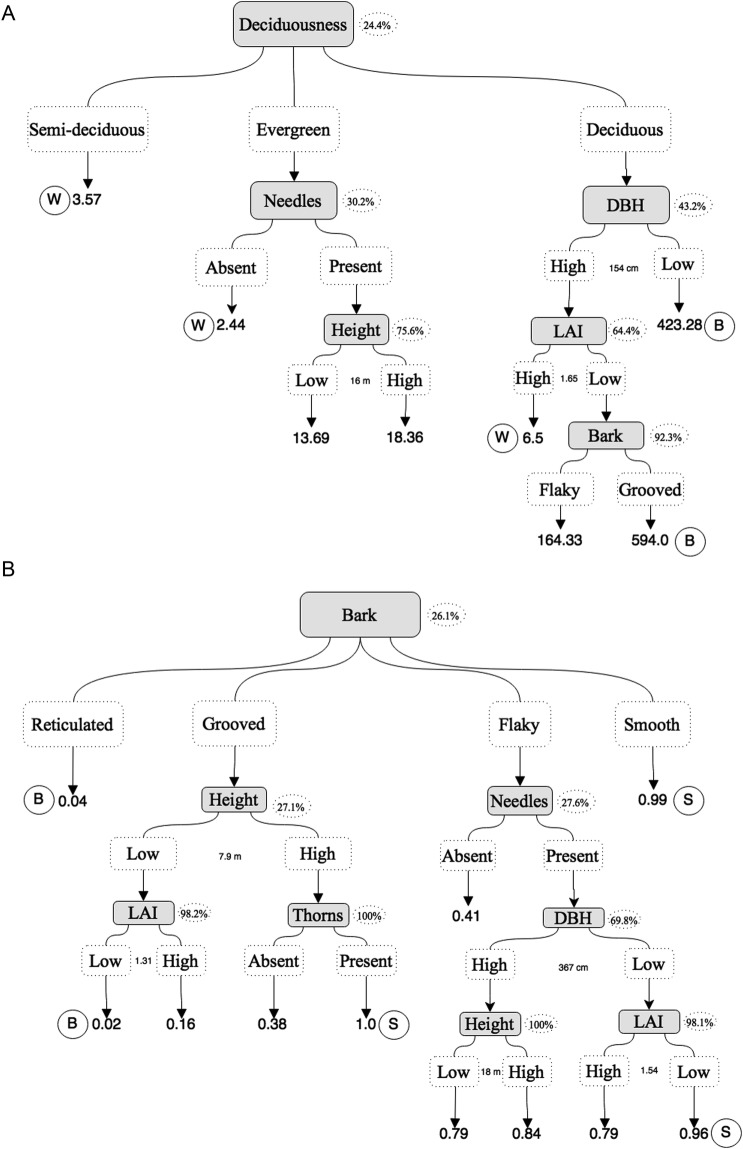
Data trees of the successive hierarchic partitions for (A) AB_abund_ and (B) AB_dst_ variances, showing the tree trait interactions. The grey rectangles represent a trait that best explained (percentage of variance explanation are showed in dotted ellipses) the variance of a group formed by a previous hierarchic partition (when preceded by an arrow). White and dotted rectangles represent the groups formed by the most explanatory trait (just above). Values between some of the white and dotted rectangles represent the median value of quantitative traits, on which the groups were formed. The values indicated by the last arrows are the mean of AB_abund_ (A) and AB_dst_ (B) of the groups formed by the trait interactions of all significant hierarchic partitions. The circles indicated the patterns of trait interactions that were named as best, worst, branches or trunk hosts (specifically B and W (A); or B and S (B)).

We found that the deciduousness of the host was the major determinant of the variance explanation in our successive hierarchic partition of the AB_abund_ (24.4 %, *z* = 3.76, *P* < 0.05): deciduous trees had higher AB_abund_, while semi-deciduous trees had lower abundance (Fig. 3A). For AB_dst_, bark type played the major role in explaining variance (26.1 %, *z* = 8.74, *P* < 0.05): trees with reticulated bark showed higher abundances of atmospheric bromeliads on branches, and trees with smooth bark possessed higher abundance on the trunk (Fig. 3B). However, the great interaction of deciduousness and bark type with other variables showed that distinct trait combinations can generate similar AB_abund_ and AB_dst_ values. For instance, semi-deciduous trees showed similar AB_abund_ than evergreen trees with needles or deciduous trees with high LAI and DBH values.

According to the functional trait interactions demonstrated by our model, the Best Hosts were those trees with (i) deciduous behaviour and DBH lower than 154 cm (AB_abund_ = 423.3 on average) and (ii) deciduous behaviour and DBH higher than 154 cm, with grooved bark and growing under an LAI lower than 1.65 (AB_abund_ = 594.0 on average; Fig. 3A). In contrast, the Worst Hosts were (i) semi-deciduous trees (AB_abund_ = 3.57 on average); (ii) evergreen trees, without needles (AB_abund_ = 2.44 on average); and (iii) deciduous trees, with a DBH higher than 154 cm, and under an LAI higher than 1.65 (AB_abund_ = 6.5 on average; Fig. 3A). Considering the position that bromeliads preferably occupy on the host, the named Branches Hosts were those trees with (i) reticulated barks (AB_dst_ = 0.04 on average) and (ii) grooved barks, height lower than 7.9 m, and under an LAI lower than 1.31 (AB_dst_ = 0.02 on average; Fig. 3B). Finally, the named Trunk Hosts were (i) trees with smooth barks (AB_dst_ = 0.99 on average); (ii) trees with flaky bark, needles, DBH lower than 367 cm and under an LAI lower than 1.54 (AB_dst_ = 0.96 on average); and (iii) trees with grooved bark, height higher than 7.9 m and without thorns (AB_dst_ = 1.0 on average; Fig. 3B).

**Figure 4.**
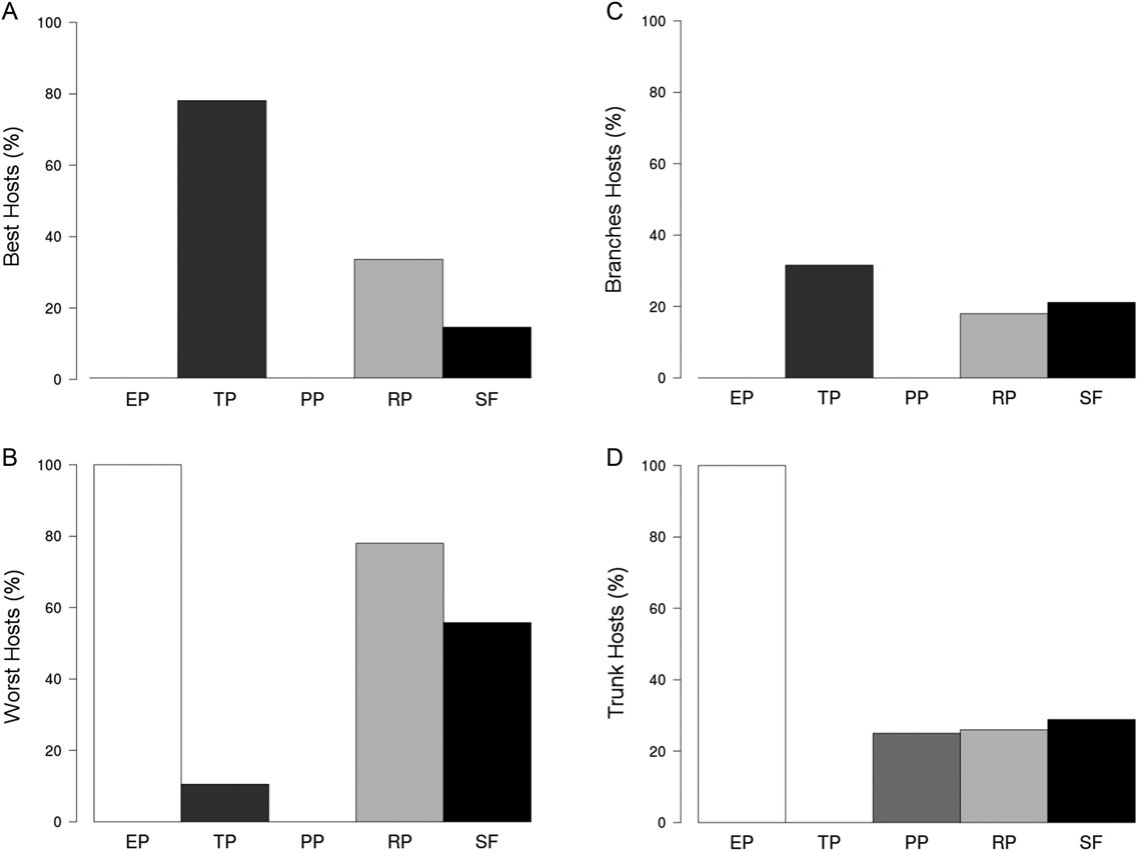
Percentage of trees assigned as (A) best, (B) worst, (C) branches and (D) trunk hosts in EP, TP, PP, RP and SF.

The *Tabebuia* spp. patch showed the higher proportions of trees assigned as Best Hosts (almost 80 %; Fig. 4A). All trees of EP, and nearly 80 % of RP, were assigned as the Worst Hosts (Fig. 4B). Nearly 30 % of host trees of TP as well as 20 % of RP and SF were assigned as Branches Hosts (Fig. 4C). All host trees of EP and nearly 30 % of PP, RP and SF were assigned as Trunk Hosts (Fig. 4D).

The first principal component of PCA using all host and no-host trees explained 47.5 % of variance of the assignment of each tree to each Host pattern, and the first principal component of PCA with only tree hosts explained 51.9 % of this variance (Table 3). Both were significantly related to AB_abund_ (*χ*^2^ = 220.97, df = 1, *P* < 0.001) and AB_dst_ (*χ*^2^ = 93.68, df = 1, *P* < 0.001; Table 3); however, the contribution of each host tree pattern showed an opposite effect on abundance and canopy distribution. While the assignment of trees to the patterns ‘Best’ and ‘Branches Hosts’ was related to the increase of abundance of atmospheric bromeliads and the relatively higher abundance on branches, the assignment of trees to the patterns of ‘Worst’ and ‘Trunk Hosts’ was correlated to the decrease of abundance and the occurrence of bromeliads on trunks (Table 3).

**Table 3.** Relationships between the AB_abund_ and AB_dst_ with the first component of PCA, performed with the presence/absence of trees assigned to each host pattern (best, worst, trunk and branches). The table also shows the percentage of the explained variance and the contribution of each host pattern on the first component of PCA. ***P < 0.001.

	PC1 scores				Explained variance (%)	Slope	*R*^2^
	Best	Worst	Trunk	Branches			
AB_abund_	−0.977	0.358	0.696	−1.154	47.5	−1.31***	0.446***
AB_dst_	−0.941	0.551	0.702	−1.325	51.9	2.57***	0.525***

**Figure 5.**
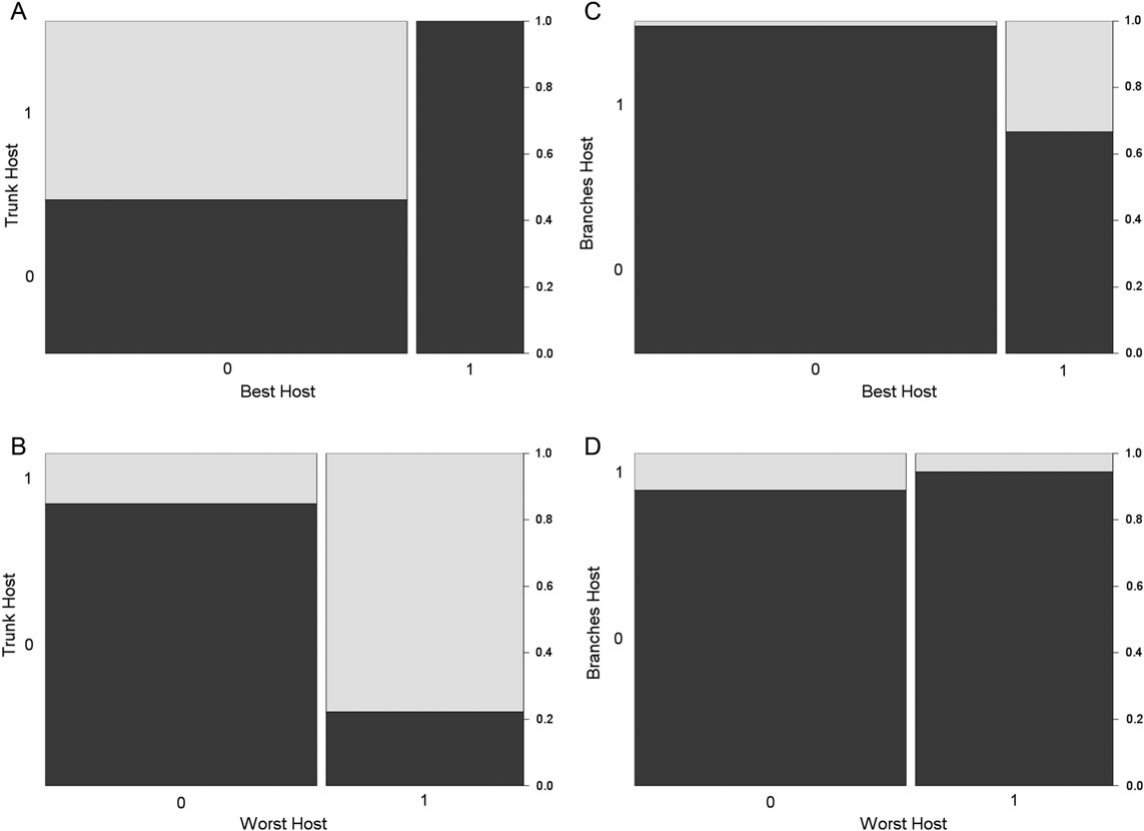
The frequencies of trees assigned as Trunk (*y*-axis of A and B) and Branches (*y*-axis of C and D) Hosts, which were also assigned as Best (*x*-axis of A and C) and Worst (*x*-axis of B and D) Hosts. 0 and 1 represent the trees that were unassigned and assigned to each specific Host pattern. The column widths represent the frequencies of trees assigned (1) and unassigned (0) as Best or Worst Hosts. The grey and black colours represent the frequencies of trees assigned (1) and unassigned (0) as Trunk or Branches Host. Only the relationship showed in (D) had no significance (*P* > 0.05).

**Figure 6.**
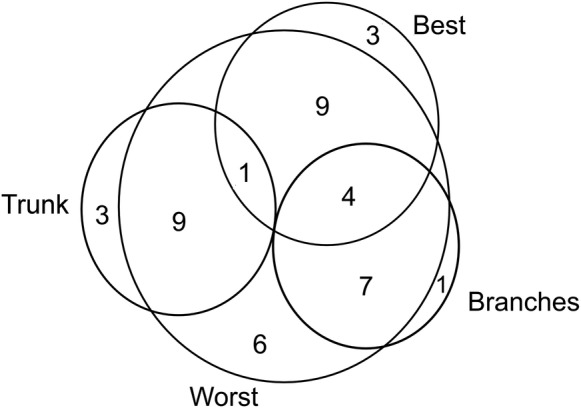
Euller diagram showing the number of tree species with individuals assigned to only one Host pattern, and the number of tree species with individuals assigned to more than one Host pattern (intersections).

We observed that trees assigned as Trunk Hosts were unlikely to be assigned as Best Hosts (*χ*^2^ = 49.88, df = 1, *P* < 0.001; Fig. 5A) and likely to be assigned as Worst Hosts (*χ*^2^ = 71.62, df = 1, *P* < 0.001; Fig. 5B), and more trees assigned as Best Hosts were likely to be also assigned as Branches Hosts than the trees unassigned to this pattern (*χ*^2^ = 31.28, df = 1, *P* < 0.001; Fig. 5C). No relationships were found among trees assigned as Worst and Branches Hosts (*χ*^2^ = 1.69, df = 1, *P* = 0.194; Fig. 5D). Many species of trees assigned as Worst Hosts also had individuals assigned to the other Host patterns (30 species; Fig. 6). Only those species of trees assigned as Branches Hosts had no individuals assigned as Trunk Hosts (Fig. 6). Each Host pattern had no >23 % of exclusive species (i.e. trees assigned to only one Host pattern; Fig 6).

## Discussion

We were able to provide evidence that stochasticity alone cannot explain the presence and abundance of atmospheric bromeliads on host trees. As observed for other epiphytes and even for *Tillandsia* spp. (Burns 2007; Reyes-García *et al*. 2008; Silva *et al*. 2010; Sáyago *et al*. 2013), we found clusters of atmospheric bromeliads on fewer tree species than expected by chance. Thus, only a few species in the pool of trees of mixed-species forests could have specific traits that can create a habitat for atmospheric bromeliad establishment, growth and reproduction, which in turn can increase their abundance. However, as recorded by Silva *et al*. (2010) for other epiphyte groups, we did not find a phylogenetic signal on the host species selection, which means that its facilitating or limiting traits are not phylogenetically conserved.

Following the small number of studies reporting host preferences of atmospheric bromeliads (e.g. Reyes-García *et al*. 2008; Flores-Palacios *et al*. 2015), our results suggest that their occurrence is related to tree traits responsible for increasing light exposition into the canopy (see Reyes-García *et al*. 2008; Einzmann *et al*. 2015), such as deciduousness, low LAI and the presence of needles (in comparison with the broad leaves of many angiosperm trees). These preferences are related to drought adaptation of some epiphytes (Einzmann *et al*. 2015), a key characteristic of atmospheric bromeliads (Benzing and Renfrow 1971; Benzing 1990; Hietz 1997; Bernal *et al*. 2005). In this study, we report impressive abundances of *Tillandsia* spp. on trees presenting such traits (>2600 individuals on a single tree) **[see Supporting Information—Fig. S5]**. This singular host preference shows not only the high reproductive capability of these plants but also their considerable ability to overcome a hard canopy filter to epiphytism (see Einzmann *et al*. 2015), comparable with the pioneer role of early successional species (e.g. Horn 1974). This comparison could also be reinforced by the higher abundance of atmospheric bromeliads on deciduous trees with lower trunk diameter (DBH)—because they are more prone to lose branches (e.g. Zimmerman and Olmsted 1992)—requiring them to complete a full life cycle in a small amount of time (Sarmento Cabral *et al*. 2015). Similar results were observed for some *Tillandsia* species (Zimmerman and Olmsted 1992), but they are not expected in other epiphytes (e.g. Yeaton and Gladstone 1982; Hietz and Hietz-Seifert 1995; Zotz 1999; Laube and Zotz 2006*a*), since they show more abundance on large trees due to the greater bark area, longer time for colonization and greater heterogeneity in microhabitats (e.g. Benzing 1990; Laube and Zotz 2007; Zotz and Schultz 2008; Woods *et al*. 2015).

The intricate interaction among the tree functional traits and architecture (e.g. Malizia 2003) can elevate or decrease its hosting capacity. For instance, in a study with *T. recurvata*, Bernal *et al*. (2005) found that in scrubland vegetation (trees with height up to 4 m), this species preferred large host trees, when comparing tree species with similar architecture. On one hand, this argument can explain the greater abundance of atmospheric bromeliads found on larger individuals of *P. elliottii* (an evergreen tree with needles; see Fig. 3A). However, it cannot explain the incredible abundance on *Tabebuia* spp., which are smaller and thinner trees. *Pinus elliottii* trees are more prone to house atmospheric bromeliads on their trunk (see Fig. 3B), because such trees are taller than *Tabebuia* spp., thus providing more trunk surface for epiphytic establishment (see Flores-Palacios and Garcia-Franco 2006; Benzing 2008; Izuddin and Webb 2015).

The bark types of trees have been associated with the epiphyte preferences of hosts (ter Steege and Cornelissen 1989; Benzing 1990; Castro Hernández *et al*. 1999; Bernal *et al*. 2005; López-Villalobos *et al*. 2008; Vergara-Torres *et al*. 2010; Wagner *et al*. 2015), but we show that for atmospheric bromeliads, this trait is more related to their distribution on host canopies rather than abundance. Trees with smooth barks house more atmospheric bromeliads on trunk, and trees with more ridged and furrowed barks house more atmospheric bromeliads on branches (Fig. 3B). Thus, these epiphytes may have a preference for smooth barks (although not directly affecting their abundance), since young branches of rough bark trees are often smoother (e.g. Everhart *et al*. 2009; Ranius *et al*. 2009). This is an interesting result for epiphytes, since smooth barks have a non-adequate surface for their attachment. However, that does not seem to be a problem for atmospheric bromeliads, which are capable of colonizing even telephone wires. Furthermore, the preference for smoother bark is probably due to its lower water-holding ability and its resulting drier micro-environment (Chomba *et al*. 2011; Wagner *et al*. 2015). Moreover, the drier conditions of the branches and their higher dew formation, as they are in an outer canopy position (see Freiberg 1997; Graham and Andrade 2004; Wagner *et al*. 2013; Woods *et al*. 2015), also explain the nesting of atmospheric bromeliads on the branches of trees with rough barks. These results suggest, once more, the preference of atmospheric bromeliads for the likely xeric conditions provided by some tree traits.

When we analysed all possible interactions of tree functional traits, we were able to discern distinct functional patterns of trees that are more prone to optimize or limit the fixation of atmospheric bromeliads. The higher similarity within distinct patterns of the ‘Best Hosts’ and ‘Branches Hosts’ (i.e. these patterns are more nested as shown in Fig. 3A and B) and the dissimilarity within the patterns of the ‘Worst Hosts’ and ‘Trunk Hosts’ (i.e. the patterns are more scattered as shown in Fig. 3A and B) are noticeable. For instance, a deciduous tree that could host large abundances of atmospheric bromeliad could also host very few individuals when presenting a high DBH or LAI. Therefore, the proportion of trees assigned as Best and Branches Hosts were lower than the other two patterns, in our sampled vegetation patches (except in TP). These results, as observed in other studies with *T. recurvata* and other epiphytes, suggest a strong host limitation of atmospheric bromeliad assemblies, on both monocultures (EP and PP) and mixed-species patches (RP and SF) (Tremblay *et al*. 1998; Bernal *et al*. 2005; Vergara-Torres *et al*. 2010; Flores-Palacios *et al*. 2015).

The balance among the proportions of Best, Worst, Trunk and Branches Hosts determines the AB_abund_ and canopy distribution in communities. The more trees are assigned as Best or Branches Hosts—or the less trees are assigned as Worst or Trunk Hosts—the more atmospheric bromeliads will be attached on the trees of a given vegetation patch and the greater the proportion of atmospheric bromeliads attaching on their branches. For instance, the TP, which showed the higher proportion of Best and Branches Hosts, showed the greatest abundance of atmospheric bromeliads, which were nested primarily on branches. In contrast, the opposite results were observed on PP, in which all trees were assigned as Worst and Trunk Hosts. On the other hand, the vegetation patches composed by multiple species of trees (RP and SF), assigned both to Best and Branches Hosts as to Worst and Trunk Hosts, showed few atmospheric bromeliads, which were almost equitably spread between trunk and branches. This result can be due to a reduction and scattering of xeric habitat on diverse forests, since their trees are able to exploit canopy space more efficiently than monocultures (Pretzsch 2014; Jucker *et al*. 2015) and could create new niches for other epiphyte types.

Given the greater amount of substrate on branches compared with trunks of many trees (Whittaker and Woodwell 1967; Ruiz-Cordova *et al*. 2014) and the differential host capacity of each of these crown partitions, our results highlight that to demonstrate host preferences of epiphytes, it is not enough to count their differential abundance on each tree. For instance, a tree could house a small amount of atmospheric bromeliads just because it has a small amount of substrate with great host capability (e.g. on its trunk), not because overall it is a bad host. Such a statement can be based on the fact that 80 % of trees with traits related to low abundance of atmospheric bromeliads (assigned as Worst Hosts) are also more prone to host them on their trunk (i.e. also assigned as Trunk Hosts), and the trees with traits related to great abundance (assigned as Best Hosts) have more chances to nest atmospheric bromeliads on their branches (i.e. also assigned as Branches Hosts), compared with those without this pattern (i.e. unassigned as Branches Hosts). Furthermore, many of tree species that were assigned as Trunk Hosts have the capability of self-pruning their branches (as *P. elliottii* and *Eucalyptus* sp.; e.g. Mäkinen and Colin 1999; Smith *et al*. 2006). So, only the atmospheric bromeliads can be fixed and increase their abundance on their trunks.

According to our results, the majority of tree species must be seen as a potentially unpleasant host for atmospheric bromeliads, since almost 90 % of tree species had individuals assigned as Worst Host. However, individual trait combinations of a tree, as well as its interaction with the neighbourhood (i.e. being shaded or changing its crown shape), can increase its host capability. In this way, individual traits of each tree are more influential in explaining differential abundance of atmospheric bromeliads. This explains why the assemblages of atmospheric bromeliads had a reduced and clumped abundance on some tree species of the diverse forests and varied widely among distinct silvicultures. Extrapolating this preference pattern to other groups of epiphytes, it becomes clear that, for conservation of epiphytic diversity, we must not only preserve the diversity of tree species but also maintain the individual abundances by species. On the other hand, species-specific traits, such as bark type or presence of thorns, may play an important role on the distribution of atmospheric bromeliads within the crown of a host tree, since no species had individuals assigned as both Branches and Trunk Hosts. So, if a tree has individual traits that make it a good host, it will be its species traits that will determine where in its crown the atmospheric bromeliads will be nested, a condition which, as stated before, will be reflected in the atmospheric bromeliad's total abundance.

## Conclusion

The assembly of atmospheric bromeliad species is not stochastic, but rather is mainly determined by a set of host trait combinations that increase the xeric conditions of epiphytic niche on different crown partitions. A balance of trees assigned to functional patterns can facilitate or limit the epiphyte's abundance and distribution. In order to detect general patterns of host preference, more studies grouping epiphyte individuals by ecological functionality, instead of using a single or all raised epiphytes species, will be essential. This approach may be more powerful and enlightening in elucidating the forces governing their assemblage.

## Sources of Funding

This research was supported by Conselho Nacional de Desenvolvimento Científico e Tecnológico (CNPQ 471756/2013-0 grant). C.J.N.C. received a scholarship from Coordenação de Aperfeiçoamento de Pessoal de Nível Superior (CAPES).

## Contributions by the Authors

D.R.R. conceived and designed the sampling. D.R.R. and J.C.D. conducted the fieldwork. C.J.N.C. analysed the data. C.J.N.C. and D.R.R. wrote the manuscript.

## Conflict of Interest Statement

None declared.

## Supporting Information

The following additional information is available in the online version of this article –

**Figure S1.** Images of all studied vegetation types.

**Figure S2.** The functional dendrogram, AB_abund_ and canopy distribution on all sampled trees.

**Figure S3.** The phylogenetic tree of all sampled tree species.

**Figure S4.** Images of all sampled atmospheric bromeliad species.

**Figure S5.** Images of atmospheric bromeliad overabundance on trees of TP (*Tabebuia* sp. grove).

**Figure S6.** The percentage of trees hosting atmospheric bromeliads in each vegetation type sampled.

**File S1.** R-scripts of null models and successive hierarchic partition.

Additional Information
