# Supplementary material for: Host trait combinations drive abundance and canopy distribution of atmospheric bromeliad assemblages
Source: AoB Plants. 2016 Feb 17;8:plw010. doi: 10.1093/aobpla/plw010 (PMC4804201; doi:10.1093/aobpla/plw010)
Supplement: Additional Information [file supp_8_plw010_index.html]

Host trait combinations drive abundance and canopy distribution of atmospheric bromeliad assemblages — Host trait combinations drive abundance and canopy distribution of atmospheric bromeliad assemblages — Additional Information 

# Host trait combinations drive abundance and canopy distribution of atmospheric bromeliad assemblages

## Additional Information

Additional Information

- Additional Information - Docx file
- Supplementary Figure 1 - docx file
- Supplementary Figure 2 - docx file
- Supplementary Figure 3 - docx file
- Supplementary Figure 4 - docx file
- Supplementary Figure 5 - docx file
- Supplementary Figure 6 - docx file
