# Supplementary material for: Host trait combinations drive abundance and canopy distribution of atmospheric bromeliad assemblages
Source: AoB Plants. 2016 Feb 17;8:plw010. doi: 10.1093/aobpla/plw010 (PMC4804201; doi:10.1093/aobpla/plw010)
Supplement: Additional Information [file supp_plw010_plw010supp.docx]

**Scritps – R software 3.1.2**

**Species richness - null models**

#### Inputs ####

setwd("~/...")

till_comun=read.csv("till_forof_tax_abund.csv",header=T,row.names=1)

library(rich)

library(vegan)

library(picante)

library(FD)

####All####

rand_till <- vector()

for(i in 1:9999){ #10000 randomizations

rand_till[i] <- rich( #calcules richness of each random draw

data.frame( #creates a data frame with each randomizations

simulate( #randomizes the data

nullmodel(till_comun,"r0_both"), #randomization keeping the total sum constant,

# cells and individuals among cells of each row are shuffled

,nsim=1)

)

)[[1]]

}

rand_till #data frame with all 10000 richness values

ric_till <- rich(till_comun)[[1]] #observed value

ric_till

table(rand_till)/sum(table(rand_till))

#### SF ####

rand_sec <- vector()

for(i in 1:9999){

rand_sec[i] <- rich(

data.frame(

simulate(

nullmodel(sec_comun,"r0_ind"),

,nsim=1)

)

)[[1]]

}

#### RP #####

rand_ref <- vector()

for(i in 1:9999){

rand_ref[i] <- rich(

data.frame(

simulate(

nullmodel(ref_comun[1:3,],"r0_ind"),

nsim=1)

)

)[[1]]

}

rand_ref

ric_ref <- rich(ref_comun)[[1]]

ric_ref

table(rand_ref)/sum(table(rand_ref))

hist(rand_ref)

#### Histograms ####

par(mfrow=c(2, 1))

hist(rand_ref,

main=NULL,

xlim=c(10,30),

col="gray",

xlab=list("Species Richness",cex=1,font=1)

) # Creates a histogram with the null model of richness

arrows(11,1500,11,0) #Observed value

quantile(rand_ref,probs=seq(0,1,0.025)) #95% IC

abline(a=NULL, b=NULL,v=21)

abline(a=NULL, b=NULL,v=26)

hist(rand_sec,

main=NULL,

xlim=c(10,30),

col="gray",

xlab=list("Species Richness",cex=1,font=1))

arrows(10,4000,10,0) #Observed value

quantile(rand_sec,probs=seq(0,1,0.025)) #95% IC

abline(a=NULL, b=NULL,v= 23)

abline(a=NULL, b=NULL,v=25)

**Functional (FDi; Cianciaruso et al. 2009) and phylogenetic (PD; Faith 1992) diversities - null models**

# Inputs ####

setwd("~/...")

till_comun=read.csv("till_forof_tax_abund.csv",header=T,row.names=1) #Species composition

tree<-read.newick(file="forof_tree_ages") #read phylogenetic relatedness among taxons

tree<-collapse.singles(tree)

forof_traits <- read.csv("forof_carac_num.csv",header=T) #Tree traits

require(phytools)

library(vegan)

library(picante)

source("read.newick.R")

####Criating a functional dendrogram####

#Standardizing data

stand <- decostand(forof_traits[,10:12], method = "standardize",na.rm=T)

stand2 <- decostand(forof_traits[,c(2:3,8:9)], method = "pa",na.rm=T)

forofstand <- data.frame(forof_traits[,4:5],stand2,stand)

## Constructing dendrogram. Podani & Schmera (2006) On dendrogram-based measures of functional diversity. Oikos 115: 179-185.

disforof <- vegdist(forofstand, method = "gower",na.rm=T,binary=T)

func_tree <- hclust(disforof, method = "average")

plot(func_tree, xlab="", main="Dendrograma", h= -1)

##### FD #####

# RP

rand_ref <- vector()

for(i in 1:9999){ #10000 randomizations

rand_ref[i]<- treedive( #calcules functional diversity of each random draw

data.frame(

simulate(

nullmodel(

till_comun,"r0_ind" # keeps row sums constant, individuals are shuffled among

#cells of each row of the matrix.

)

,nsim=1

)

),func_tree,match.force = F

)[[4]]

}

summary(rand_ref)

fd_ref <- treedive(till_comun,func_tree)[[4]] #Observed functional diversity

fd_ref

table(rand_ref/sum(table(rand_ref)))

par(mfrow=c(2, 1))

#Criating the histogram

hist(rand_ref,

main=NULL,

xlim=c(2.5,5.5),

ylim=c(0,3000),

col="gray",

xlab=list("FD",cex=1,font=1)

)

arrows(fd_ref,2000,fd_ref,0) #Observed FD

quantile(rand_ref,probs=seq(0,1,0.025)) #95%IC

abline(a=NULL, b=NULL,4000 ,3.603617) #2.5%

abline(a=NULL, b=NULL,4000 ,4.825473) #97.5%

# SF

rand_sec <- vector()

for(i in 1:9999){

rand_sec[i]<- treedive(

data.frame(

simulate(

nullmodel(

till_comun,"r0_ind"

)

,nsim=1

)

),func_tree,match.force = F

)[[5]]

}

summary(rand_sec)

fd_sec <- treedive(till_comun,func_tree,match.force = F)[[5]]

fd_sec

table(rand_sec/sum(table(rand_sec)))

hist(rand_sec,

main=NULL,

xlim=c(1,6),

ylim=c(0,3000),

col="gray",

xlab=list("FD",cex=1,font=1)

)

arrows(fd_sec,2000,fd_sec,0)

quantile(rand_sec,probs=seq(0,1,0.025))

abline(a=NULL, b=NULL,4000 ,3.694306)

abline(a=NULL, b=NULL,4000 ,4.914890)

##### PD #####

#RP

rand_ref <- till_comun[4,]

for(i in 1:9999){ #Creates a null model with randomizations

rand_ref[i,]<- data.frame(

simulate(

nullmodel(

till_comun,"r0_ind"

),nsim=1

)

)[4,]

}

rand_refpd<- data.frame(pd(rand_ref,tree)) #Calculates the Phylogenetic Diversity of all randomizations

names(rand_refpd)

refpd <- rand_refpd$PD[-1]

par(mfrow=c(2, 1))

hist(refpd,

main=NULL,

col="gray",

xlab=list("PD",cex=1,font=1)

)

arrows(rand_refpd$PD[1],1500,rand_refpd$PD[1],0) #Observed PD

quantile(refpd,probs=seq(0,1,0.025))

abline(a=NULL, b=NULL,v=3376.374)

abline(a=NULL, b=NULL,v=4597.168)

#SF

rand_sec <- till_comun[5,]

for(i in 1:9999){

rand_sec[i,]<- data.frame(

simulate(

nullmodel(

till_comun,"r0_ind"

),nsim=1

)

)[5,]

}

rand_secpd<- data.frame(pd(rand_sec,tree))

secpd <- rand_secpd$PD[-1]

hist(secpd,

main=NULL,

col="gray",

xlab=list("PD",cex=1,font=1)

)

arrows(rand_secpd$PD[1],1500,rand_secpd$PD[1],0)

quantile(secpd,probs=seq(0,1,0.025))

abline(a=NULL, b=NULL,v=3522.735)

abline(a=NULL, b=NULL,v=4737.443)

**Atmospheric bromeliad abundance - successive hierarchic partitions**

#### input ####

setwd("~/...")

index_tree <- read.csv("tree_index_abundtill_rel.csv",header=T)

library(vegan)

library(nlme)

library(gplots)

library(candisc)

library(lsmeans)

library(glmulti)

library(hier.part)

par(mar=c(5,7,2,2) + 0.1)

#####

index_tree <- index_tree[,-12]

names(index_tree)

levels(index_tree[,10]) <- c(0,1)

levels(index_tree[,12]) <- c(0,1)

levels(index_tree[,13]) <- c(0,1)

##### Standardization #####

names(index_tree[,8:10])

stand <- decostand(index_tree[,5:7], method = "standardize",na.rm=T)

stand2 <- data.frame(as.factor(index_tree[,10]),as.factor(index_tree[,12]),

as.factor(index_tree[,13]))

treestand <- data.frame(index_tree[,c(8:9,11)],stand2,stand,index_tree$abund_till,index_tree$abund_rel)

names(treestand)[10:11] <- c("abund_till","abund_rel")

names(treestand)[4:6] <- c("descam_forof","espinho_forof","aciculas_forof")

##### First Hierarchical Partition ####

summary(treestand)

env<- treestand[,-c(1,10:11)] #environment variables

summary(env)

hier.part(treestand$abund_till,env, family="poisson",gof="logLik") #Perform the hierarchical partition

rand.hp(treestand$abund_till,env, family="poisson",gof="logLik",num.reps=1000)$Iprobs #Randomization for 1000 draws to test significance

##### Subsetting_total #####

abund_per <- subset(treestand,decid_forof == "perene" )

abund_dec <- subset(treestand,decid_forof == "decidua" )

abund_semi <- subset(treestand,decid_forof == "semi" )

##### Perene #####

summary(abund_per)

env<- abund_per[,c(3,4,6:9)]

summary(env)

hier.part(abund_per$abund_till,env, family="poisson",gof="logLik")

rand.hp(abund_per$abund_till,env, family="poisson",gof="logLik",num.reps=1000)$Iprobs

##### Subsetting_perene #####

abund_acic_s <- subset(abund_per,aciculas_forof == 1 )

abund_acic_n <- subset(abund_per,aciculas_forof == 0 )

##### Acicula_presente #####

summary(abund_acic_s)

env<- abund_acic_s[,c(7:9)]

summary(env)

hier.part(abund_acic_s$abund_till,env, family="poisson",gof="logLik")

rand.hp(abund_acic_s$abund_till,env, family="poisson",gof="logLik",num.reps=1000)$Iprobs

##### Subsetting_acic_s #####

abund_alt_a <- subset(abund_acic_s,alt_forof > median)

abund_alt_b <- subset(abund_acic_s,alt_forof <= median)

##### Altura alta #####

summary(abund_alt_a)

env<- abund_alt_a[,c(7:8)]

summary(env)

hier.part(abund_alt_a$abund_till,env, family="poisson",gof="logLik")

rand.hp(abund_alt_a$abund_till,env, family="poisson",gof="logLik",num.reps=1000)$Iprobs

##### Altura baixa #####

summary(abund_alt_b)

env<- abund_alt_b[,c(7:8)]

summary(env)

hier.part(abund_alt_b$abund_till,env, family="poisson",gof="logLik")

rand.hp(abund_alt_b$abund_till,env, family="poisson",gof="logLik",num.reps=1000)$Iprobs

##### Acicula_ausente #####

summary(abund_acic_n)

env<- abund_acic_n[,c(7:9)]

summary(env)

hier.part(abund_acic_n$abund_till,env, family="poisson",gof="logLik")

rand.hp(abund_acic_n$abund_till,env, family="poisson",gof="logLik",num.reps=1000)$Iprobs

##### Semi-decídua #####

summary(abund_semi)

env<- abund_semi[,c(3,5,7:9)]

summary(env)

hier.part(abund_semi$abund_till,env, family="poisson",gof="logLik")

rand.hp(abund_semi$abund_till,env, family="poisson",gof="logLik",num.reps=1000)$Iprobs

##### Decídua #####

summary(abund_dec)

env<- abund_dec[,c(3,5,7:9)]

summary(env)

hier.part(abund_dec$abund_till,env, family="poisson",gof="logLik")

rand.hp(abund_dec$abund_till,env, family="poisson",gof="logLik",num.reps=1000)$Iprobs

##### Subsetting_dec #####

abund_dbh_a <- subset(abund_dec,diam_forof > median) #Utilizada a mediana

abund_dbh_b <- subset(abund_dec,diam_forof <= median) #Utilizada a mediana

##### DBH_alto ####

summary(abund_dbh_a)

env<- abund_dbh_a[,c(3,5,7,9)]

summary(env)

hier.part(abund_dbh_a$abund_till,env, family="poisson",gof="logLik")

rand.hp(abund_dbh_a$abund_till,env, family="poisson",gof="logLik",num.reps=1000)$Iprobs

##### Subsetting abund_dbh_a #####

abund_LAI_a <- subset(abund_dbh_a,LAI_forof > median) #Utilizada a mediana

abund_LAI_b <- subset(abund_dbh_a,LAI_forof <= median) #Utilizada a mediana

##### LAI_alto ####

summary(abund_LAI_a)

env<- abund_LAI_a[,c(3,5,9)]

summary(env)

hier.part(abund_LAI_a$abund_till,env, family="poisson",gof="logLik")

rand.hp(abund_LAI_a$abund_till,env, family="poisson",gof="logLik",num.reps=1000)$Iprobs

##### LAI_baixo ####

summary(abund_LAI_b)

env<- abund_LAI_b[,c(3,9)]

summary(env)

hier.part(abund_LAI_b$abund_till,env, family="poisson",gof="logLik")

rand.hp(abund_LAI_b$abund_till,env, family="poisson",gof="logLik",num.reps=1000)$Iprobs

##### Subsetting abund_LAI_b #####

abund_casc_esc <- subset(abund_LAI_b,tipocasca_forof == "escamosa" )

abund_casc_sulc <- subset(abund_LAI_b,tipocasca_forof == "sulcada" )

##### DBH_baixo ####

summary(abund_dbh_b)

env<- abund_dbh_b[,c(3,7,9)]

summary(env)

hier.part(abund_dbh_b$abund_till,env, family="poisson",gof="logLik")

rand.hp(abund_dbh_b$abund_till,env, family="poisson",gof="logLik",num.reps=1000)$Iprobs

##### LAI_baixo ####

summary(abund_LAI_b)

env<- abund_LAI_b[,c(3,5,8:9)]

summary(env)

hier.part(abund_LAI_b$abund_till,env, family="poisson",gof="logLik")

rand.hp(abund_LAI_b$abund_till,env, family="poisson",gof="logLik",num.reps=1000)$Iprobs

##### Subsetting_LAI_b #####

abund_casc_ret <- subset(abund_LAI_b,tipocasca_forof == "reticulada" )

abund_casc_esc <- subset(abund_LAI_b,tipocasca_forof == "escamosa" )

abund_casc_sulc <- subset(abund_LAI_b,tipocasca_forof == "sulcada" )

##### Casca reticulada ####

summary(abund_casc_ret)

env<- abund_casc_ret[,c(5,8:9)]

summary(env)

hier.part(abund_casc_ret$abund_till,env, family="poisson",gof="logLik")

rand.hp(abund_casc_ret$abund_till,env, family="poisson",gof="logLik",num.reps=1000)$Iprobs

##### Casca escamosa ####

summary(abund_casc_esc)

env<- abund_casc_esc[,c(8:9)]

summary(env)

hier.part(abund_casc_esc$abund_till,env, family="poisson",gof="logLik")

rand.hp(abund_casc_esc$abund_till,env, family="poisson",gof="logLik",num.reps=1000)$Iprobs

##### Casca sulcada ####

summary(abund_casc_sulc)

env<- abund_casc_sulc[,c(5,8:9)]

summary(env)

hier.part(abund_casc_sulc$abund_till,env, family="poisson",gof="logLik")

rand.hp(abund_casc_sulc$abund_till,env, family="poisson",gof="logLik",num.reps=1000)$Iprobs

##### abund_casc_sulc #####

abund_esp_s <- subset(abund_casc_sulc,espinho_forof == 1)

abund_esp_n <- subset(abund_casc_sulc,espinho_forof == 0)

##### Espinho ausente ####

summary(abund_esp_n)

env<- abund_esp_n[,c(8:9)]

summary(env)

hier.part(abund_esp_n$abund_till,env, family="poisson",gof="logLik")

rand.hp(abund_esp_n$abund_till,env, family="poisson",gof="logLik",num.reps=1000)$Iprobs

**Atmospheric bromeliad canopy distribution - successive hierarchic partitions**

##### input #####

setwd("~/...")

index_tree <- read.csv("tree_index_abundtill_rel.csv",header=T)

library(vegan)

library(nlme)

library(gplots)

library(candisc)

library(lsmeans)

library(glmulti)

library(hier.part)

par(mar=c(5,7,2,2) + 0.1)

#####

index_tree <- index_tree[,-12]

names(index_tree)

levels(index_tree[,10]) <- c(0,1)

levels(index_tree[,12]) <- c(0,1)

levels(index_tree[,13]) <- c(0,1)

index_tree <- index_tree[!(is.na(index_tree$abund_rel) | index_tree$abund_rel==""), ]

fust <- index_tree$abund_fuste/index_tree$abund_till

index_tree <- cbind2(index_tree,fust)

names(index_tree)[27] <- "fust"

View(index_tree)

##### Standardization #####

names(index_tree[,8:10])

stand <- decostand(index_tree[,5:7], method = "standardize",na.rm=T)

stand2 <- data.frame(as.factor(index_tree[,10]),as.factor(index_tree[,12]),

as.factor(index_tree[,13]))

treestand <- data.frame(index_tree[,c(8:9,11)],stand2,stand,index_tree$abund_till,index_tree$abund_rel,index_tree$fust)

names(treestand)[10:12] <- c("abund_till","abund_rel","fust")

names(treestand)[4:6] <- c("descam_forof","espinho_forof","aciculas_forof")

##### First Hierarchical Partition ####

summary(treestand)

env<- treestand[,-c(1,10:12)] #environment variables

summary(env)

hier.part(treestand$fust,env, family="binomial"(link="logit"), gof="logLik") #Perform the hierarchical partition

rand.hp(treestand$fust,env, family="binomial"(link="logit"), gof="logLik",num.reps=1000)$Iprobs #Randomization for 1000 draws to test significance

##### Subsetting_total #####

abund_casc_esc <- subset(treestand,tipocasca_forof == "escamosa")

abund_casc_lisa <- subset(treestand,tipocasca_forof == "lisa" |tipocasca_forof == "laminada")

abund_casc_ret <- subset(treestand,tipocasca_forof == "reticulada")

abund_casc_sulc <- subset(treestand,tipocasca_forof == "sulcada")

##### Lisa #####

summary(abund_casc_lisa)

env<- abund_casc_lisa[,c(2,4,7:9)]

summary(env)

hier.part(abund_casc_lisa$fust,env, family="binomial"(link="logit"),gof="logLik")

rand.hp(abund_casc_lisa$fust,env, family="binomial"(link="logit"),gof="logLik",num.reps=1000)$Iprobs

##### Escamosa #####

summary(abund_casc_esc)

env<- abund_casc_esc[,c(2,6:9)]

summary(env)

hier.part(abund_casc_esc$fust,env, family="binomial"(link="logit"),gof="logLik")

rand.hp(abund_casc_esc$fust,env, family="binomial"(link="logit"),gof="logLik",num.reps=1000)$Iprobs

##### Subsetting abund_casc_esc #####

abund_acic_s <- subset(abund_casc_esc,aciculas_forof == "1")

abund_acic_n <- subset(abund_casc_esc,aciculas_forof == "0")

##### Acículas S #####

summary(abund_acic_s)

env<- abund_acic_s[,c(7:9)]

summary(env)

hier.part(abund_acic_s$fust,env, family="binomial"(link="logit"),gof="logLik")

rand.hp(abund_acic_s$fust,env, family="binomial"(link="logit"),gof="logLik",num.reps=1000)$Iprobs

##### Subsetting abund_acic_s #####

abund_dbh_a <- subset(abund_acic_s,diam_forof > median)

abund_dbh_b <- subset(abund_acic_s,diam_forof <= median)

##### DBH alto #####

mean(abund_dbh_b$abund_rel)

summary(abund_dbh_a)

env<- abund_dbh_a[,c(7,9)]

summary(env)

hier.part(abund_dbh_a$fust,env, family="binomial"(link="logit"),gof="logLik")

rand.hp(abund_dbh_a$fust,env, family="binomial"(link="logit"),gof="logLik",num.reps=1000)$Iprobs

##### Subsetting abund_dbh_a #####

abund_alt_a <- subset(abund_dbh_a,alt_forof > median)

abund_alt_b <- subset(abund_dbh_a,alt_forof <= median)

##### DBH baixo #####

summary(abund_dbh_b)

env<- abund_dbh_b[,c(7,9)]

summary(env)

hier.part(abund_dbh_b$fust,env, family="binomial"(link="logit"),gof="logLik")

rand.hp(abund_dbh_b$fust,env, family="binomial"(link="logit"),gof="logLik",num.reps=1000)$Iprobs

##### Subsetting abund_dbh_b #####

abund_lai_a <- subset(abund_dbh_b,LAI_forof > median)

abund_lai_b <- subset(abund_dbh_b,LAI_forof <= median)

##### Acículas N #####

summary(abund_acic_n)

env<- abund_acic_n[,c(2,7:9)]

summary(env)

hier.part(abund_acic_n$fust,env, family="binomial"(link="logit"),gof="logLik")

rand.hp(abund_acic_n$fust,env, family="binomial"(link="logit"),gof="logLik",num.reps=1000)$Iprobs

##### Sulcada #####

summary(abund_casc_sulc)

env<- abund_casc_sulc[,c(2,5,7:9)]

summary(env)

hier.part(abund_casc_sulc$fust,env, family="binomial"(link="logit"),gof="logLik")

rand.hp(abund_casc_sulc$fust,env, family="binomial"(link="logit"),gof="logLik",num.reps=1000)$Iprobs

##### Subsetting abund_casc_sulc #####

abund_alt_a <- subset(abund_casc_sulc,alt_forof > median)

abund_alt_b <- subset(abund_casc_sulc,alt_forof <= median)

##### alt alto #####

summary(abund_alt_a)

env<- abund_alt_a[,c(2,5,7:8)]

summary(env)

hier.part(abund_alt_a$fust,env, family="binomial"(link="logit"),gof="logLik")

rand.hp(abund_alt_a$fust,env, family="binomial"(link="logit"),gof="logLik",num.reps=1000)$Iprobs

##### Subsetting abund_alt_a #####

abund_esp_s <- subset(abund_alt_a,espinho_forof == "1")

abund_esp_n <- subset(abund_alt_a,espinho_forof == "0")

##### esp_s #####

summary(abund_esp_s)

##### esp_n #####

summary(abund_esp_n)

env<- abund_esp_n[,c(2,7:8)]

summary(env)

hier.part(abund_esp_n$fust,env, family="binomial"(link="logit"),gof="logLik")

rand.hp(abund_esp_n$fust,env, family="binomial"(link="logit"),gof="logLik",num.reps=1000)$Iprobs

##### alt baixa #####

summary(abund_alt_b)

env<- abund_alt_b[,c(7:8)]

summary(env)

hier.part(abund_alt_b$fust,env, family="binomial"(link="logit"),gof="logLik")

rand.hp(abund_alt_b$fust,env, family="binomial"(link="logit"),gof="logLik",num.reps=1000)$Iprobs

##### Subsetting abund_alt_b #####

abund_lai_a <- subset(abund_alt_b,LAI_forof > median)

abund_lai_b <- subset(abund_alt_b,LAI_forof <= median)

##### lai alto #####

summary(abund_lai_a)

##### lai baixo #####

summary(abund_lai_b)

##### Reticulada #####

summary(abund_casc_sulc)

env<- abund_casc_ret[,c(5,7:9)]

summary(env)

hier.part(abund_casc_ret$fust,env, family="binomial"(link="logit"),gof="logLik")

rand.hp(abund_casc_ret$fust,env, family="binomial"(link="logit"),gof="logLik",num.reps=1000)$Iprobs
