# Supplementary material for: Host trait combinations drive abundance and canopy distribution of atmospheric bromeliad assemblages
Source: AoB Plants. 2016 Feb 17;8:plw010. doi: 10.1093/aobpla/plw010 (PMC4804201; doi:10.1093/aobpla/plw010)
Supplement: Additional Information [file supp_plw010_plw010supp_fig1.docx]

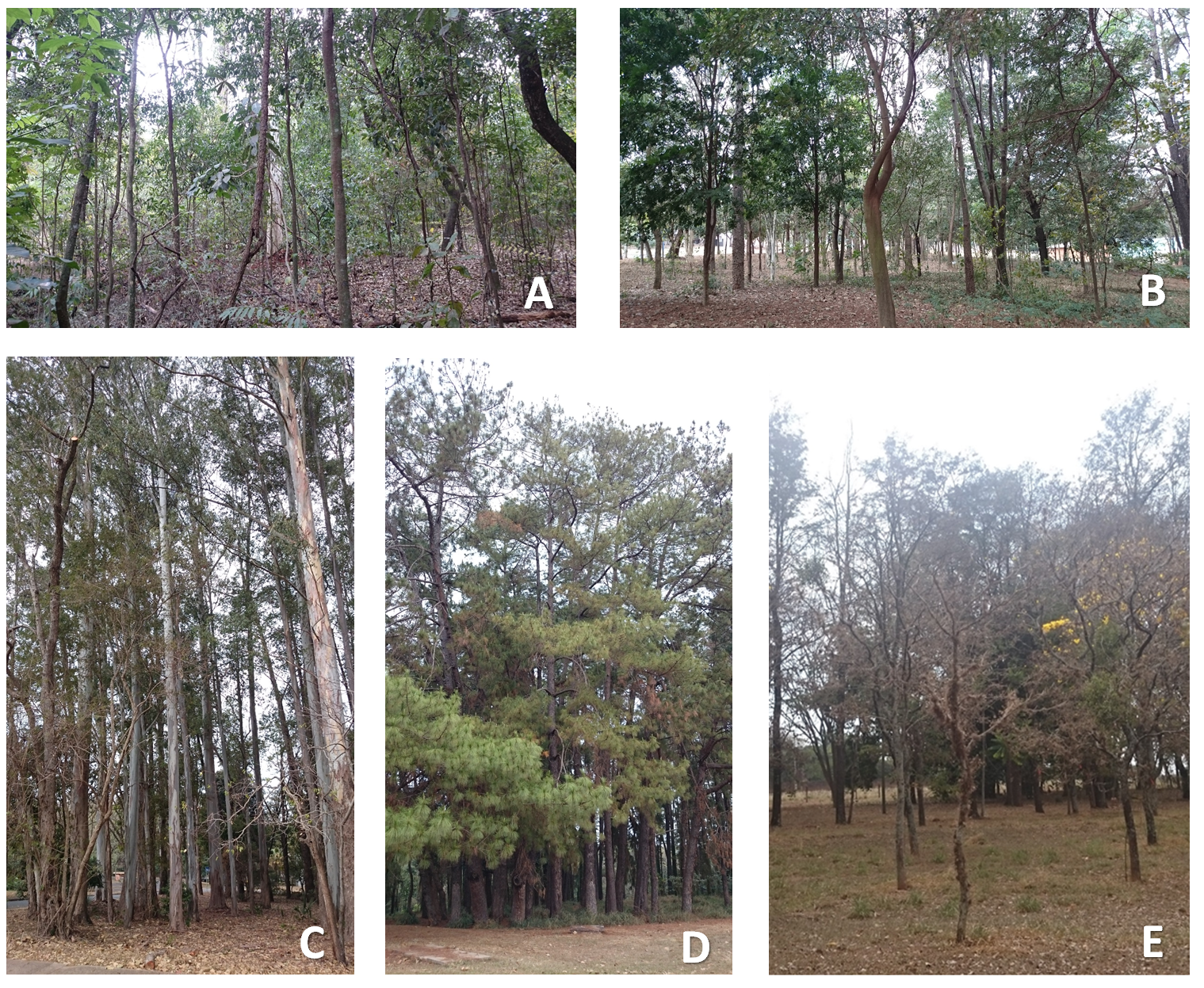


**Figure S1**. The studied vegetation types. A - Secondary semi deciduous forest (SF); B - semi deciduous forest reforestation patch (RP); C - *Eucalyptus* sp. patch (EP); D - *Pinus elliottii* patch (PP) and E - *Tabebuia* sp. (Bignoniaceae) grove (TP).
