# Supplementary material for: Host trait combinations drive abundance and canopy distribution of atmospheric bromeliad assemblages
Source: AoB Plants. 2016 Feb 17;8:plw010. doi: 10.1093/aobpla/plw010 (PMC4804201; doi:10.1093/aobpla/plw010)
Supplement: Additional Information [file supp_plw010_plw010supp_fig2.docx]

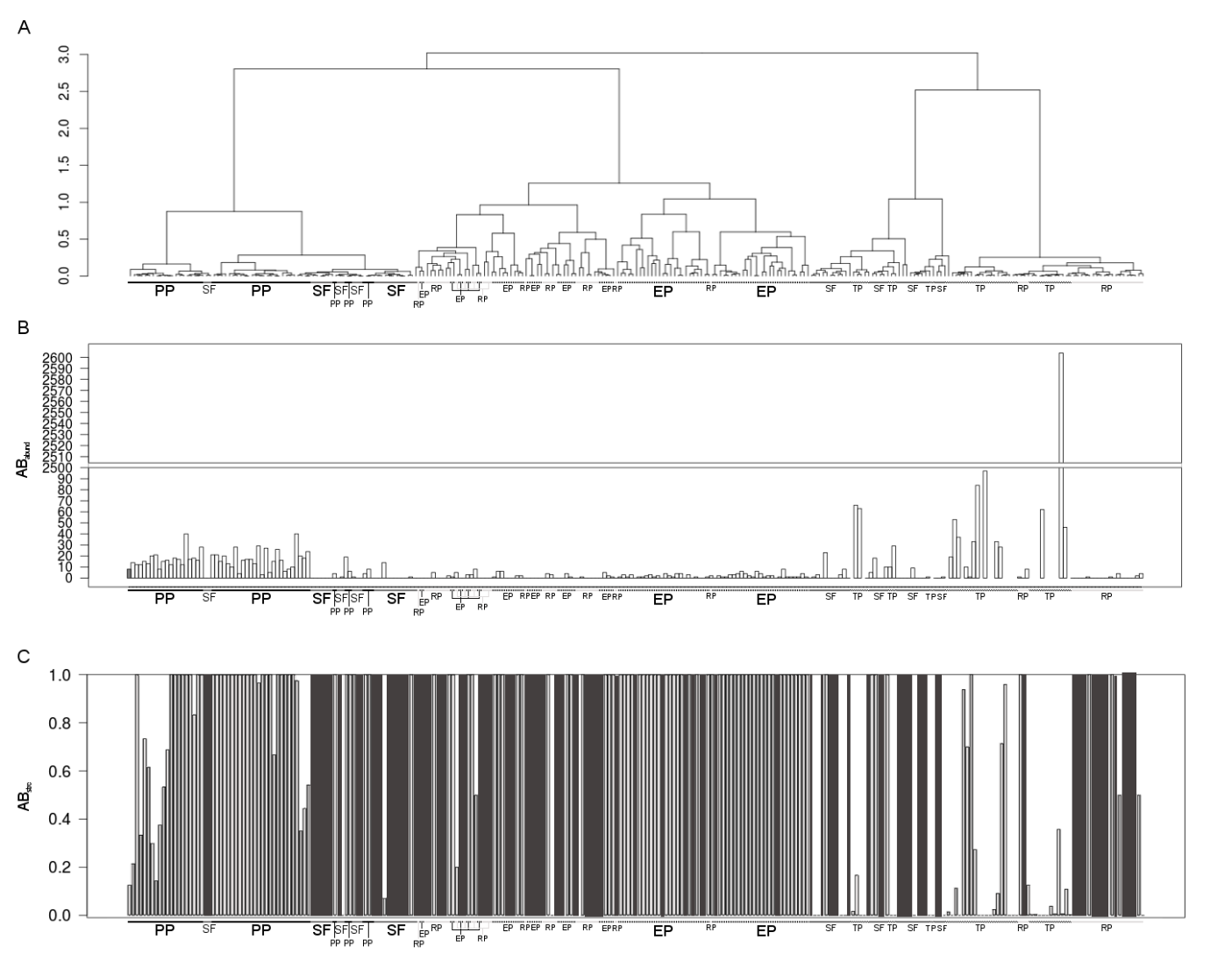


**Figure S2**. (A) Functional dendogram, (B) atmospheric bromeliad abundance, and (C) atmospheric bromeliad canopy distribution of all trees of studied vegetation patches. SF - secondary semi deciduous forest; RP - semi deciduous forest reforestation patch; EP - *Eucalyptus* sp. patch; PP - *Pinus elliottii* patch and TP - *Tabebuia* sp. (Bignoniaceae) grove. The dark gray areas in C represent trees were no atmospheric bromeliad were found.
