# Supplementary material for: Host trait combinations drive abundance and canopy distribution of atmospheric bromeliad assemblages
Source: AoB Plants. 2016 Feb 17;8:plw010. doi: 10.1093/aobpla/plw010 (PMC4804201; doi:10.1093/aobpla/plw010)
Supplement: Additional Information [file supp_plw010_plw010supp_fig3.docx]

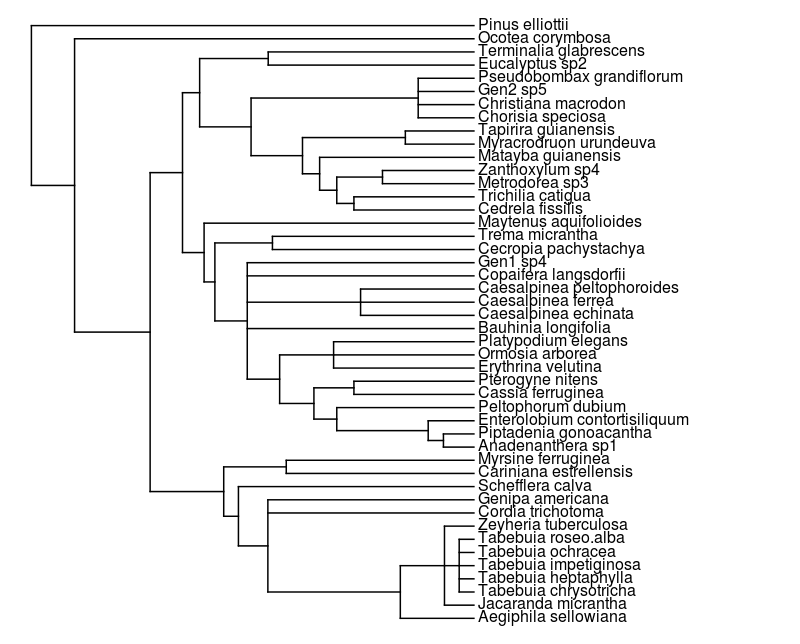


**Figure S3**. Phylogenetic relationships of the tree hosts species accounting for all studied situations.
