# Supplementary material for: Host trait combinations drive abundance and canopy distribution of atmospheric bromeliad assemblages
Source: AoB Plants. 2016 Feb 17;8:plw010. doi: 10.1093/aobpla/plw010 (PMC4804201; doi:10.1093/aobpla/plw010)
Supplement: Additional Information [file supp_plw010_plw010supp_fig4.docx]

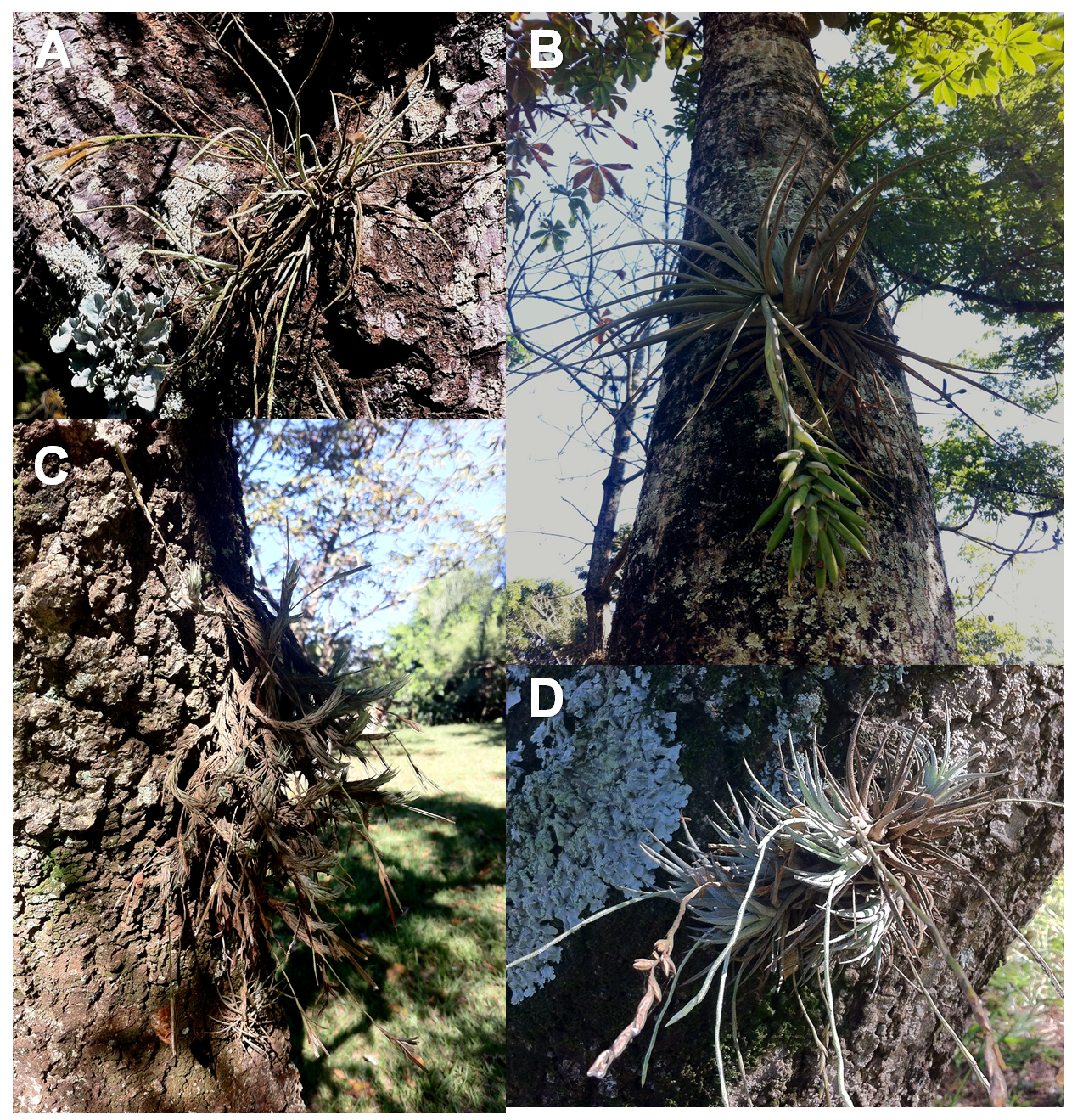


**Figure S4**. The sampled atmospheric bromeliads species in our studied sites. A - *Tillandsia* *recurvata* (L.) L.; B - *Tillandsia pohliana* Mez; C - *Tillandsia tricholepsis* Baker and D - *Tillandsia loliacea* Mart. ex Schult.f
