# Supplementary material for: Host trait combinations drive abundance and canopy distribution of atmospheric bromeliad assemblages
Source: AoB Plants. 2016 Feb 17;8:plw010. doi: 10.1093/aobpla/plw010 (PMC4804201; doi:10.1093/aobpla/plw010)
Supplement: Additional Information [file supp_plw010_plw010supp_fig5.docx]

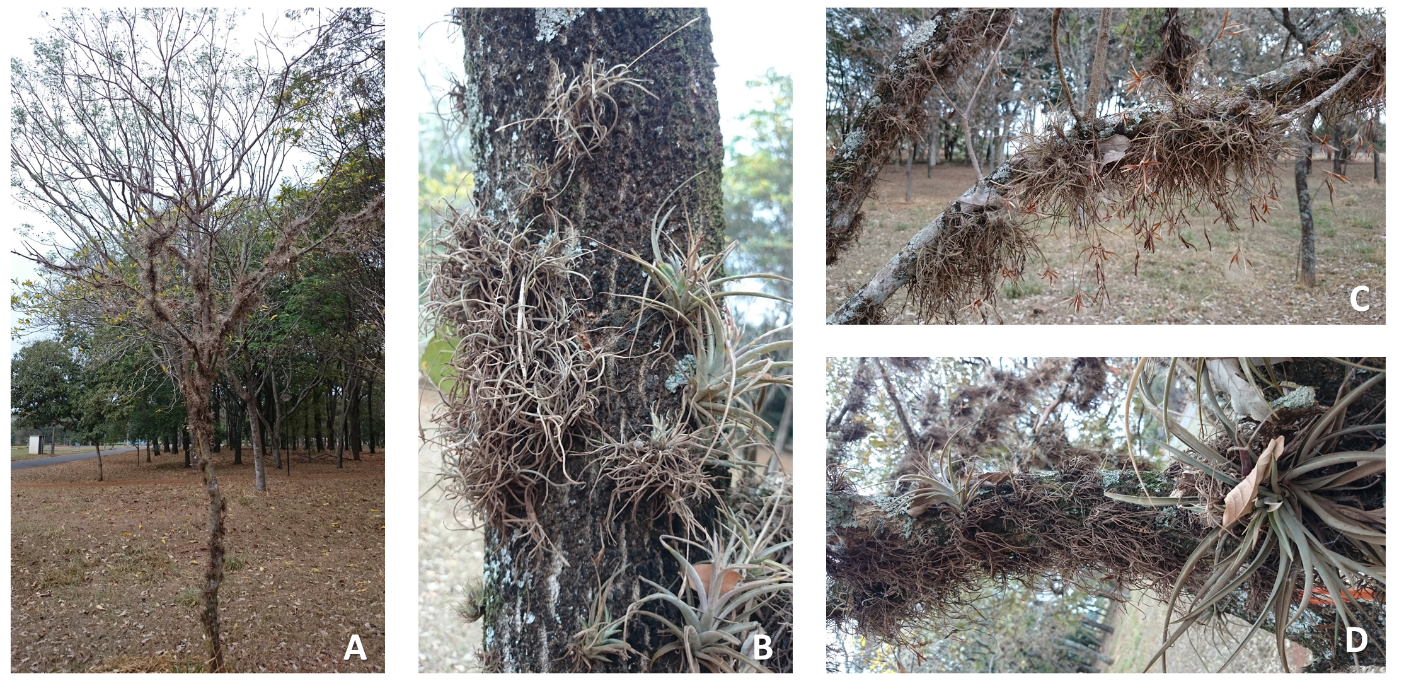


**Figure S5**. A - Overabundance of *Tillandsia* spp. on *Tabebuia roseo-alba* (Bignoniaceae). B – Overabundance of *T. recurvata* and *T. pohliana* in the trunk of *T. roseo-alba*. C – Overabundance of *T. recurvata* and *T. pohliana* in the branches of *T. roseo-alba* and D – overabundance of *T. recurvata* and *T. pohliana* in the trunk of *Tabebuia crisotrycha* (Bignoniaceae).
