# Supplementary figures and images for: Host trait combinations drive abundance and canopy distribution of atmospheric bromeliad assemblages
Source: AoB Plants. 2016 Feb 17;8:plw010. doi: 10.1093/aobpla/plw010 (PMC4804201; doi:10.1093/aobpla/plw010)

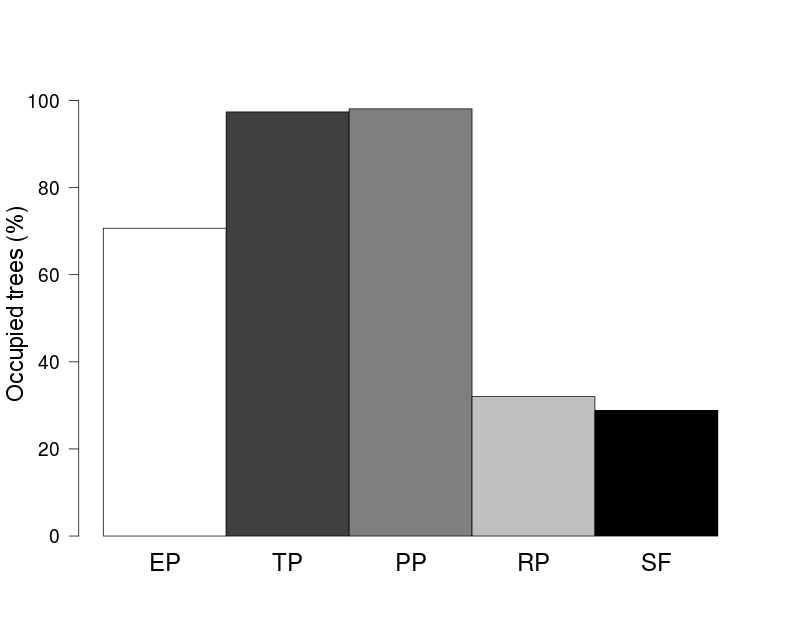


**Figure S6**. Percentages of tree hosting atmospheric bromeliads.

Supplement: Additional Information [file supp_plw010_plw010supp_fig6.docx]
